# Stress and its influence on reproduction in pigs: a review

**DOI:** 10.1186/1751-0147-50-48

**Published:** 2008-12-10

**Authors:** Stig Einarsson, Ylva Brandt, Nils Lundeheim, Andrzej Madej

**Affiliations:** 1 Division of Reproduction, Department of Clinical Sciences, Faculty of Veterinary Medicine and Animal Science, Swedish University of Agricultural Sciences, Box 7054, SE-75007 Uppsala, Sweden; 2 Department of Animal Breeding and Genetics, Faculty of Veterinary Medicine and Animal Science, Swedish University of Agricultural Sciences, Box 7023, SE-75007 Uppsala, Sweden; 3 Department of Anatomy, Physiology and Biochemistry, Faculty of Veterinary Medicine and Animal Science, Swedish University of Agricultural Sciences, Box 7011, SE-75007 Uppsala, Sweden

## Abstract

The manifestations of stress, defined as a biological response to an event that the individual perceives as a threat to its homeostasis, are commonly linked to enhanced activity of the hypothalamo-pituitary-adrenal (HPA) axis and the activation of the sympathetic adreno-medullary (SA) system. Activation of the HPA system results in the secretion of peptides from the hypothalamus, principally corticotropin releasing hormone (CRH), which stimulates the release of adrenocorticotropic hormone (ACTH) and beta-endorphin. ACTH induces the secretion of corticosteroids from the adrenal cortex, which can be seen in pigs exposed to acute physical and/or psychological stressors. The present paper is a review of studies on the influence of stressors on reproduction in pigs. The effects of stress on reproduction depend on the critical timing of stress, the genetic predisposition to stress, and the type of stress. The effect of stress on reproduction is also influenced by the duration of the responses induced by various stressors. Prolonged or chronic stress usually results in inhibition of reproduction, while the effects of transient or acute stress in certain cases is stimulatory (e.g. anoestrus), but in most cases is of impairment for reproduction. Most sensitive of the reproductive process are ovulation, expression of sexual behaviour and implantation of the embryo, since they are directly controlled by the neuroendocrine system.

## Background

### Definition of stress

Stress can be defined in many ways, but one of the most accepted definitions is a biological response to an event that the individual perceives as a threat to its homeostasis [1]. Perception of stressful stimuli leads e.g. to activation of the hypothalamo-pituitary-adrenal (HPA) system, which in turn results in the release of a variety of peptides, principally corticotropin releasing hormone (CRH) and vasopressin from the hypothalamus [2]. CRH stimulates the release of adrenocorticotrophic hormone (ACTH) and other proopiomelanocortin (POMC) derived peptides, such as β-endorphin from the anterior lobe of the pituitary gland. ACTH acts on the adrenal glands and causes secretion of glucocorticoid hormones, e.g. cortisol. ACTH also causes the release of other hormones from the adrenal glands, e.g. progesterone, possibly prostaglandin F_2α _metabolite [3] and even inhibin α [4]. Stress also involves the activation of the sympathetic nervous system and the adrenal medulla. This causes the release of catecholamines e.g. adrenaline and noradrenaline into the bloodstream, leading to an increase in the glucose supply by accelerating the degradation of glycogen in the liver [5]. The glucocorticoids also stimulate lipolysis and gluconeogenesis (the conversion of amino acids to glucose), which leads to an increased metabolism that promotes the ability to cope with stress [6].

### Assessment of stress

There are many difficulties involved in evaluation of, and comparing how different types of stress affect animal welfare in general, especially in long-term stressful situations. Stress response can be assessed by determining the activation of the HPA-axis and/or the sympathetic adreno-medullay (SA) system, by measuring the level of secreted peptides in the peripheral blood plasma, urine, cerebrospinal fluid, saliva, etc. Behavioural responses such as heart rate, blood pressure and stereotypical behaviour, as well as the effects on the immune response can also be used for the assessment of stress response [7-10]. However meaningful evaluation of these responses requires a detailed knowledge of the normal physiological and behaviour patterns of the animal studied, because the response to stress is influenced by several factors such as the metabolic condition, health status, age, sexual maturity. The stress response is equally dependant on the nature, intensity and duration of the stressful event. Also, there is a large individual variation between pigs in their ability to cope with stress and the fact that each stressor both has a non-specific effect (stimulation of the HPA-axis) and a specific effect. The latter is the "biological target" for the stressor. Heat stress, for example, leads to hyperthermia of the sow, food deprivation to a catabolic metabolism. For this reason, there is no stressor that can be used as a standard stressor to evaluate stress response in general. The specific response of each stressor has to be evaluated separately.

The present paper is a review of some studies on the influence of stressors on reproduction in pigs in terms of environment, management and housing like lactation, weaning, transportation/relocation, grouping. Also, in a series of experimental studies from our own group, stress was simulated with fasting or repeated ACTH treatments for approximately 48 hours.

### Temperature and humidity

#### Boars

Pigs have a low capacity for increased sweating when the ambient temperature increases e.g. from 23 to 34°C, which contributes to the close relationship between environmental temperature and scrotal and testicular temperatures during such periods [11]. Lowered fertility and/or lowered total sperm counts (TSC) and decreased ejaculate volume have been found in boars during or shortly after the warm summer period in several countries with temperate areas such as Europe and North America [12-15]. In tropical areas such as Thailand temperature has a significant negative effect on both the ejaculate volume, TSC and the morphology of the spermatozoa [16-18].

In recent years, a new housing system, called an evaporative cooling system (EVAP) or tunnel ventilation, has been introduced to improve the microclimate for livestock production in regions with hot climate [16]. The EVAP system is a closed housing system, which aims to reduce the temperature via humidification process. In a comprehensive Swedish/Thai study sperm production and sperm morphology was recorded in boars kept in EVAP system vs conventional housing system (CONV) [16-18]. Temperature and humidity were recorded on a daily basis during one calender year. There was a higher diurnal variation and range over the year for both temperature and humidity in the CONV system compared to the EVAP system. The average maximum temperature was lower and the average minimum humidity was higher in the EVAP system, than in the CONV system. There was no overall difference in sperm production and sperm morphology between boars kept in the CONV and the EVAP housing systems. However, during parts of the year, differences between systems in sperm production and sperm morphology were observed. Elevated temperature had a significant negative effect on both the ejaculate volume and TSC in both housing systems. Elevated humidity had a significant negative effect on both the ejaculate volume and TSC in the EVAP system. To further minimize the negative impact of high temperature and high humidity on sperm production of boars under tropical conditions, further investigations on economically competetive technologies that can decrease both temperature and humidity are needed.

In several experiments the effect of elevated temperature (in terms of heat stress) on the spermatogenesis of boars has been investigated using a climatic chamber. McNitt and First [19] found a reduced TSC and an increased percentage of abnormal spermatozoa around two weeks after placing boars in a climatic chamber at 33°C and 50% RH (% relative humidity) for 72 hours. Exposure of boars for 35°C and 40% RH for 100 hours resulted in decreased sperm quality, in terms of an increased percentage of abnormal spermatozoa; the ejaculate volume and TSC per ejaculate remained unaltered [20]. Additionally, local heating of the scrotum has caused similar disturbances in spermatogenesis [21,22]. In most studies, an increased proportion of abnormal spermatozoa has been found after heat treatment, but the results vary among boars, and are also related to the different regimes for causing heat stress [20,23-25]. An acute rise in rectal temperature related to the detrimental effects on the testes was observed in some heat exposed boars [24]. This indicates that the stress, imposed by elevated ambient temperature, may not be of the same magnitude for all boars.

#### Gilts and sows

Heat stress has been reported to reduce implantation and impair embryo development in pig. Edwards et al [26] found that gilts are more sensitive to heat stress before day 15 of pregnancy, than during days 15–30 post-breeding. Omtvedt et al. [27] illustrated a greater reduction in the number of viable embryos among gilts exposed to elevated temperatures during days 8–16 post-breeding than days 0–8, indicating that the time of implantation would be the most sensitive stage of pregnancy to stress.

High ambient temperature leading to heat stress has been associated with seasonal infertility. This is especially true not only in tropical areas, for example in Thailand, where the temperature exceeds 30°C for several months of the year [28], but also in temperate areas, for example in countries in northern Europe and the USA [29-31]. In the referred comprehensive Swedish/Thai field study performed in Thailand, there was a significant influence by climate, maximum temperature and heat index on litter size, farrowing rate and weaning to first service interval [28]. Several experimental studies have been performed using two or more temperature levels kept constant over the day at a low or moderate relative humidity [32-34]. In these studies a prolonged weaning-to-service interval was recorded at the high temperatures; this prolongation has been reported to associate partly with a reduced appetite [33,35]. Suriyasomboon et al. [36] collected daily climatic (temperature, relative humidity) data within a number of herds in Thailand during one calender year. They found a seasonal variation in reproductive performance of the sows, but there was no indication that high temperature and humidity at previous weaning/mating or at farrowing had negative effects on litter size.

#### Short lactation and weaning

Several technical improvements have made it possible to carefully study e.g. follicles, cysts and corpora lutea in the ovaries [37] and their relationship to husbandry practice like short lactation, weaning and housing. Before introduction of transrectal ultrasonography [38,39] the only possible ways to study the ovaries in pigs were either to inspect and make morphological examinations after slaughter [40], or to carry out laborious laparoscopic examinations [41,42].

Kunavongkrit [41] performed comparative clinical and endocrine studies of sows, when all piglets were removed from their dams within 12 hours of farrowing, called "zero-weaning". Using repeated laparoscopic examination, he could endocrinologically compare sows that showed oestrus within 2 weeks after parturition and developed ovarian cysts (anovulatory) with sows that ovulated regularly [43]. None of the anovulatory sows had a well defined pre-ovulatory LH surge. The peripheral plasma concentration of cortisol was significantly higher in the anovulatory than in the ovulatory sows [44], indicating that elevated cortisol might be one factor inhibiting the LH surge. It is noteworthy that some pairs of full sibs behaved similarly following zero-weaning. Hereditary factors may thus play a role in the development of ovarian cysts in pigs.

A husbandry practice like weaning seems to be a stressful stimulus for the sow. Thus, weaning after five weeks of lactation resulted in a concomitant increase of plasma concentrations of cortisol and β-endorphin [45,46]. Sows in good nutritional status, which failed to resume oestrous activity after weaning had significantly higher plasma concentrations of cortisol and β-endorphin and lower LH concentrations around weaning than sows that resumed oestrous activity [47].

#### Transportation/relocation

There are a few reports of acute stressors stimulating various aspects of reproduction. Thus, Hughes [48] showed that transport of female pigs advanced the onset of puberty. Combined clinical and endocrinological studies have been performed on the effect of transportation and/or relocation on gilts with delayed puberty [49] and anoestrous sows [50,51]. Approximately 75% of the gilts with delayed puberty and 75% of the anoestrous sows showed ovulatory oestrus within one week, after approximately one hour of transportation. The pulse frequency of LH increased significantly right after transportation, and increased oestradiol-17β concentrations were detected in utero-ovarian vein plasma 8–16 hours after transportation, or 24–36 hours earlier than in jugular blood. The increased LH activity following transportation indicates that the hypothalamus and/or the higher brain centre of these animals responded by increased activity to this short-term stress. In a subsequent study, Dalin et al. [52] showed that the plasma concentrations of both cortisol and catecholamines are elevated in pigs that are transported. Transportation/relocation might therefore act as a positive stressor on hypothalamus in anoestrous female pigs.

The release of catecholamines occurs very rapidly after stress exposure [52,53], and the plasma half-life of the amines is extremely short, which makes it difficult to determine an accurate and yet comparable timing in different treatments. Moreover, the validity of measurement of venous blood plasma noradrenaline has been questioned, because regional or general differences in sympathetic activity may not be reflected accurately in venous blood samples taken from a single site [9]. Therefore, cortisol is still considered a more valid blood parameter reflecting stress in clinical studies of adult pigs.

#### Managements

The management procedures in modern pig production include a number of events, which might act as stressors on the animals, e.g. high stocking densities, barren environments, transportation, poor or aggressive human-animal interactions and heat stress. Due to welfare consideration, systems with loose-housed sows instead of system with crates/stalls for the sows have become common, at least for non-lactating sows. These systems have several advantages: i.e. the animals have possibility to perform their natural behaviour. However, there are factors in these systems causing problems. In systems with loose-housed sows, the number of sows are in most cases much higher than in groups formed in the wild. The female pigs form family units of one or several sows and their offsprings [54], and the individuals in the family unit avoid contact with other unfamiliar sows [55]. Therefore, in wild pigs (Sus scrofa) confrontation between unfamiliar female pigs happens very rarely. In commercial group housing systems, however, mixing of unfamiliar sows is difficult to avoid. A drawback with the group-housing system is also the difficulty to avoid regrouping. In most lactation units the sows are housed individually, and grouping of unfamiliar sows usually takes place at least once after weaning. A new social grouping in a limited space results in aggressive behaviour among the animals. Depending on the social status of the animal, i.e. dominant or subordinate, different individuals have various capabilities in competing situations such as feeding and watering. An animal that does not cope with these situations may have a reduced well-being and impaired reproductive performance. High ranked sows in oestrus will also mount submissive sows, and will rise to periods of stress [56]. Elevated stress levels in a newly formed small group of sows may persist for approximately two days until a ranking order is established among the animals [57], and even continue for additional 10–12 days in large groups of sows [58].

#### Herd investigations

Peltoniemi et al. [59] found that rebreeding was performed more often after an irregular oestrus-to-oestrus interval, i.e. 25–37 days in group housed sows than in sows kept in individual stalls, particularly during summer and autumn. Kongsted [60] described the impaired reproduction in group housed sows as a growing problem in many herds. Group housing conditions resulted in fewer born piglets per litter when comparing with individually housed sows [61,62]. Moreover, Pedersen et al. [63] reported that group housing may result in the impairment of heat detection and response to boar stimulation. In a comprehensive study by Karlén et al. [64] on welfare including reproduction of gestating sows in conventional stalls and large groups on deep litter, sows on deep litter had a higher return to oestrus after mating (13% versus 7 %, p < 0.01) and there was a tendency (p = 0.06) for higher salivary cortisol concentrations in week one of gestation in deep litter sows (the sows were recently mated when they entered respective treatment). However, measurement of cortisol in saliva is not always a reliable method to monitor stress in pigs [65]. Altogether, the reproductive parameters recorded show that sows in stalls weaned the equivalent of 39 more piglets per 100 mated sows than sows in large groups. The results suggest that sows in large groups on deep litter faced greater welfare challenges in the early stages of gestation, all possibly a consequence of aggression. In contrast, sows in stalls faced greater welfare challenges later in gestation based on a higher incidence of feet and leg problems. In addition, the evidence of stereotypical behaviour may indicate some disadvantages for sows kept in stalls for the whole gestation. On the other hand, Cassar et al. [66], investigating mixed-parity sows assigned to be housed individually or in groups of 15 from the time of insemination and for 5 weeks, found no effect of grouping per se on farrowing rate or subsequent litter sizes.

Recently an excellent study was presented by Munsterhjelm et al. [67] investigating in 12 replicates of 40 sows the effects of housing on pregnancy rate 28 days post-service, early disruption of pregnancy and behaviour. Half of the dry sows were kept in stalls, and half were group-housed on 5 m^2 ^deep litter per sow. Stall-housing was associated with signs of stress caused by the lack of exercise and a rootable substrate. Behavioural indicators proposed a lower welfare level in stalled animals compared with group-housed ones. This type of stress – or the level thereof – did affect reproduction in terms of weaning-to-oestrus interval, rebreeding rate and irregular rebreeding (% of rebreedings) significantly less than the social stress experienced by group-housed sows.

#### Experimental studies

Soede et al. [68] investigated hormone patterns, oestrus, ovulation and early embryo development in multiparous sows that had been tethered during lactation, and after weaning were either tethered by neck chain, or individually housed in a pen of approximately 6 m^2^. The registration of above mentioned parameters started at two months after weaning (the sows were not mated in the post-wening oestrus, and altrenogest treatment was given for oestrous synchronization). The profiles of oestradiol-17β, LH and progesterone around oestrus were similar for both treatment groups, while the duration of oestrus was shorter in the tethered sows. The sows were euthanised day 5 after ovulation; ovulation rate, fertilization rate, embryo development and embryo diversity were similar for the two groups. The results from this study indicate that sows that had been tethered during lactation, and were housed loose or were tethered again at weaning, differed in stereotypic behaviour and in duration of oestrus, without effects on reproductive hormones, two months later.

In a recent study Soede et al. [69] investigated the effect of repeated stress treatments during the follicular phase and early pregnancy on reproductive performance of gilts. All gilts were housed individually. The animals given a stressful treatment were grouped for half an hour at the start of the treatment and during the treatment period nose-sling and an unpredictable feeding scheme were applied regularly. Despite this rather harsh treatment, saliva cortisol levels and even the reproductive performance were not disturbed. A possible reason for lack of changes in cortisol levels might be related to findings that measurement of cortisol in saliva is not always an accurate method to monitor stress in pigs [65]. It is also plausible that gilts used by Soede et al. [69] were particularly resistant to imposed negative handling treatment.

### Stress simulated by ACTH treatment during pro-oestrus/oestrus

High doses of ACTH (daily intramuscular injections) administered for several days during the follicular phase during the oestrous cycle (from days 16–18 until the end of oestrus) [70] caused a delay in the onset of oestrus and development of cystic follicles in sows. In an earlier study Liptrap [71] had clearly demonstrated that cystic follicles were induced in adrenal intact sows by treatment with high doses of ACTH, but not in adrenalectomized sows. These results indicate that the disruption of the ovarian function was caused by cortisol, released from the adrenal glands by exogenous ACTH.

To simulate "stressful" events after weaning and around pro-oestrus/oestrus like aggressive behaviour among sows after grouping/mixing, competetive situations at feeding and drinking, riding etc. repeated injections of small doses of ACTH were given for approximately 48 hours to multiparous healthy sows in a series of experiments (performed by our own research group). Follicular growth and ovulation were monitored using ultrasonography. Blood samples were collected frequently before, during and after treatment.

#### ACTH given for 48 hours during pro-oestrus

Onset of oestrus was predicted based upon the individual progesterone profile and the time for reaching a progesterone concentration < 8 nmol/L [72]. The interval between the time when progesterone reached a level of < 8 nmol/L and onset of oestrus was prolonged with 2.5 days in the ACTH-cycles vs the control-cycles. At onset of oestrus, the follicles were larger in the ACTH sows than in the control sows. In some sows, ovulation was disturbed.

#### Administration of ACTH for up to 48 hours from onset of oestrus to ovulation

Cortisol and progesterone were significantly elevated in jugular blood samples [3,10]. The display of signs of standing oestrus went away more rapidly after ovulation due to the rise in progesterone concentrations, but there was no effect on the time of ovulation.

When insemination was performed once, approximately 18 hours before ovulation and the sows were anesthetized/killed at 4–8 hours after ovulation, there was a tendency towards a larger number of spermatozoa in the UTJ and oviduct among the ACTH sows compared with the control sows [10,73]. The majority of spermatozoa seemed to have intact membranes. A majority of sows in the ACTH group had moderately to exaggerated amounts of mucus in the intraluminal environment of the sperm reservoir, which might be due to the higher progesterone concentrations than the control group [10,74].

In a subsequent study the sows were killed at 48 or 60 hours after ovulation and retrieval rate of embryos and oocytes and their localization were investigated [10,75]. Fewer oocytes/embryos were retrieved from the ACTH group than from the control group (51% vs 81%, P < 0.05). There was also a tendency towards faster embryo transportation to the uterus in the ACTH group, perhaps due to the high progesterone concentrations during treatment.

### Stress induced by food deprivation or ACTH treatment for 48 hours during two periods of the early pregnancy

Multiparous sows were deprived of food (FD), but had free access of drinking water, or were treated with ACTH for 48 hours after ovulation. There was a delayed embryonic cleavage rate and a decreased number of spermatozoa attached to zona pellucida (ZP) in FD sows, reflecting a change in the oviductal environment [76,77]. Postovulatory food deprivation also delayed the oviductal ova transport rate [6,78], which may be due to a prostaglandin-associated prolonged contraction of the isthmic muscle [79,80]. ACTH had no effect on the oviductal transport rate of the embryos, but a negative effect on the embryo development in terms of cleavage rate and a lower number of spermatozoa attached to the ZP compared with the controls [6,81,82].

Food deprivation during days 10 and 11 of pregnancy did not cause any effect on embryo recovery rate at day 17 of pregnancy [45,83]. When the treatments (FD or ACTH) were performed during days 13 and 14 of pregnancy, there was a significant effect on endocrine status of the sows during the treatment period. Both FD and ACTH sows had increased levels of cortisol, but only FD sows had increased levels of progesterone and PGF_2α_-metabolite [6,83]. There were no effects on total number of foetuses or foetal survival rate, observed at day 30 of pregnancy [6,84,85]. However, the progesterone concentration of the allantoic fluid of FD sows at day 30 was increased compared with controls and also correlated to the size of the placentas. ACTH stimulation caused a two-day delay in the increase of plasma oestrone concentration, seen at day 19 of pregnancy in control sows. In gilts, injected with hydrocortisone acetate at 12-hour intervals from day 11 to day 20 of pregnancy, a two-day delay of the peak of oestrone sulphate was also observed [86,87]. The absence of effects on the embryo survival at day 30 might be due to: (1) large individual variation in the ability to cope with stress in combination with relatively small groups, (2) the degree of stress was not severe enough, or the sows were not exposed to the treatments for a long enough period of time. It must also be taken into account that the sows used in the study were in very good nutritional and physical condition, and as reproduction is the most important activity of any species, the capacity to compensate for the effects of stress is very well developed.

## Conclusion

The effects of stress on reproduction depend on the critical timing of stress, the genetic predisposition to stress, and the type of stress. The effect of stress on reproduction is also influenced by the duration of the responses induced by various stressors. Prolonged or chronic stress usually results in inhibition of reproduction, while the effects of transient or acute stress in certain cases is stimulatory (e.g. anoestrus), but in most cases is of impairment for reproduction. Most sensitive of the reproductive process are ovulation, expression of sexual behaviour and implantation of the embryo, since they are directly controlled by the neuroendocrine system.

## Competing interests

The authors declare that they have no competing interests.

## Authors' contributions

Stig Einarsson has been involved in most of the studies performed in Sweden and reviewed in this manuscript. He has also drafted the major part of this review. Ylva Brandt has performed some of the studies reviewed in this manuscript, and helped to draft the manuscript. Nils Lundeheim has participated in several of the studies and also made the statistics in many of the studies reviewed in this manuscript. He has helped to draft the manuscript. Andrzej Madej has been involved in many of the studies reviewed in this manuscript, and also helped to draft the manuscript.
